# Impact of Operative Position on Outcomes of Proximal Femoral Nailing: A Comparison Between Supine on a Traction Table and Lateral Decubitus

**DOI:** 10.7759/cureus.108284

**Published:** 2026-05-05

**Authors:** Navdeep Singh Keer, Balu Ravi, Loveneesh Krishna, Raaghav Rai Verma, Ishaan Siwach, Utkarsh Jain

**Affiliations:** 1 Sports Injury, Vardhman Mahavir Medical College and Safdarjung Hospital, Delhi, IND; 2 Orthopaedics, The Royal Wolverhampton NHS Trust, Wolverhampton, GBR; 3 Orthopaedics, Vardhman Mahavir Medical College and Safdarjung Hospital, Delhi, IND; 4 Orthopaedics, Pandit Bhagwat Dayal Sharma Post Graduate Institute of Medical Sciences, Rohtak, IND

**Keywords:** cephalomedullary nail, functional outcome, harris hip score, intertrochanteric fractures, lateral decubitus position, proximal femoral nailing, radiographic union, supine position, tip apex distance, traction table

## Abstract

Introduction

Unstable intertrochanteric fractures are common in older adults and are best stabilised with cephalomedullary nails. Surgeons favour either a supine position on a traction table or a lateral decubitus position on a standard table, yet evidence comparing their clinical value is limited and often conflicting. The traction table may simplify reduction and imaging but introduces set-up delays and post-related complications; lateral positioning avoids the post but relies on manual traction. This study aims to determine whether patient position meaningfully impacts intraoperative, radiographic, and functional outcomes in proximal femoral nailing using a single implant and uniform perioperative protocol.

Materials and methods

This is a prospective observational study done at a tertiary trauma centre over 18 months. Sixty adults with AO Foundation/Orthopaedic Trauma Association (AO/OTA) 31-A2/A3 fractures were consecutively enrolled after informed consent and further allocated to either Group A (supine on traction table, n = 30) or Group B (lateral decubitus on standard table, n = 30) based on equipment availability. All underwent closed reduction and proximal femoral nail antirotation-II (PFNA-II) fixation by the same surgical team with identical anaesthesia and rehabilitation protocols. Outcomes measured were set-up time, operative time, ease of fluoroscopy/traction (4-point Likert scale), tip-apex distance (TAD), Cleveland-Bosworth quadrant, collodiaphyseal angle, four-week fracture gap, signs of union till 24 weeks by modified Radiographic Union Score for Hip (RUSH), and Harris Hip Score (HHS). Radiographs were read by two blinded observers. Standard parametric/non-parametric tests were used (α = 0.05).

Results

All patients completed a 24-week follow-up. Supine positioning required a longer set-up but a shorter operative time. Fluoroscopy and traction were rated “easy” more often in the supine group. Blood loss was similar between groups. Supine cases achieved lower TAD, more frequent central/central or inferior/central blade placement, and more neutral-to-valgus alignment. The early (four-week) fracture gap was smaller, and mobilisation occurred sooner in the supine cohort. Despite a larger early fracture gap, the lateral cohort showed earlier callus formation at 8-12 weeks, and by 16 to 24 weeks, union rates were comparable between the two groups. HHS trajectories were similar at baseline and four weeks, showed a transient lateral advantage at 12 weeks, and converged by 24 weeks. One transient sciatic neurapraxia occurred in the supine group; no deep infection, implant failure, or reoperation was recorded.

Conclusion

Supine traction-table positioning improves intraoperative control and early rehabilitation, while lateral decubitus achieves similar union and 24-week function without specialised equipment. Position can be selected to match resources and patient needs, provided reduction quality and implant placement are prioritised.

## Introduction

Intertrochanteric fractures are extracapsular injuries between the greater and lesser trochanters and constitute a large proportion of hip fractures in older adults. Their incidence rises steeply with age and osteoporosis and is associated with excess short-term morbidity and mortality from immobility-related complications. In younger patients, high-energy trauma is the usual mechanism [1-3].

Fracture stability guides treatment strategy. Patterns with an intact posteromedial buttress and sufficient lateral wall are considered stable and tolerate controlled collapse, whereas comminution, reverse obliquity, or subtrochanteric extension render a fracture unstable and prone to varus or rotational failure [4]. Sliding hip screw devices remain dependable for stable injuries, yet multiple trials now favour intramedullary fixation for unstable configurations because the shorter lever arm reduces implant load and permits earlier full weight-bearing [5].

Proximal femoral nail antirotation-II (PFNA-II) is widely adopted for such fractures. Its helical blade compacts cancellous bone, improving purchase in osteoporotic femoral heads, and the angular stability of the nail-blade junction limits medialisation and cut-out [6]. Although implant design is largely standardised, surgical positioning varies. Most surgeons use a traction table with the patient supine: traction applied through a perineal post maintains length while fluoroscopy is obtained in anteroposterior (AP) and lateral planes. The alternative is lateral decubitus positioning on a radiolucent table, which allows a direct entry point, frees the contralateral limb, and eliminates post-related pressure complications [7].

Existing literature is inconclusive on whether one position confers measurable advantages. Retrospective studies differ in fracture stability metrics, implant type, or outcome reporting. Although several single-centre series have addressed operative time, implant placement, or union rates, only a handful of prospective comparative studies or meta-analyses directly evaluate the two positions in a uniform clinical protocol [8].

The aim of this study is to address this gap by prospectively comparing supine fixation on a traction table with lateral decubitus fixation for unstable intertrochanteric fractures using the same implant and to clarify whether patient position meaningfully influences technical or clinical results. To our knowledge, it is the first study to couple detailed intraoperative metrics with serial radiographic and functional outcomes for these two positions, providing evidence directly applicable to everyday practice in similar settings.

## Materials and methods

**Figure 1:**
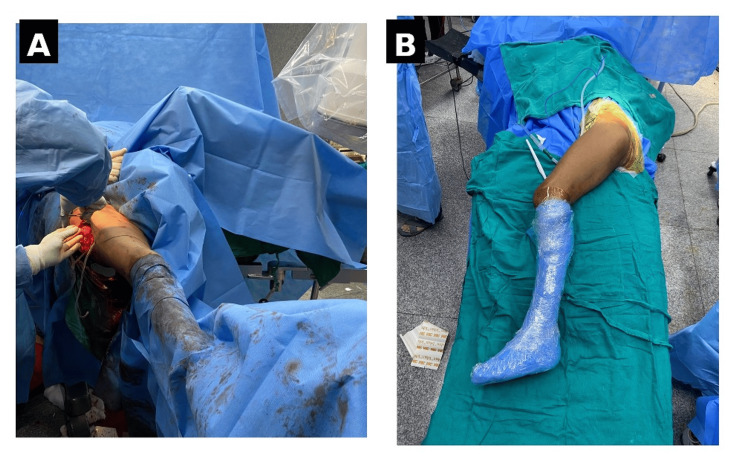
Intraoperative patient positioning. The figure illustrates the practical differences in operating room set-up that form the study’s intraoperative metrics. In the supine position on a traction table (A), counter-traction and traction are provided by the post/boot system, enabling stable reduction and unobstructed fluoroscopic arcs. In the lateral decubitus position (B), counter-traction is achieved with body supports and manual traction, allowing easy access to the proximal femur and freedom of the knee for distal locking manoeuvres.

We conducted a prospective, two-arm comparative cohort over 18 months at a tertiary trauma centre, with prior ethics approval. Adults with unstable intertrochanteric fractures were consecutively enrolled after informed consent. Allocation to Group A (supine position on a traction table) or Group B (lateral decubitus position on a radiolucent table) was determined by the availability of the respective operative set-up on the day of surgery (Figures 1A, 1B). Surgeons and patients were not blinded; radiographic measurements were performed by two independent observers blinded to group allocation, and “ease” ratings had an independent second rater.

Patient selection

Unstable fractures were defined as AO Foundation/Orthopaedic Trauma Association (AO/OTA) 31-A2 or 31-A3 patterns, or Boyd-Griffin type III. Inclusion criteria include age ≥ 18 years, AO/OTA 31-A2 or 31-A3 (unstable intertrochanteric fracture), suitability for PFNA-II, surgery within 10 days of injury, and ability to provide consent and attend follow-up. Exclusion criteria include pathologic fracture, periprosthetic fracture, associated ipsilateral femoral shaft or neck fracture, polytrauma precluding either position, prior ipsilateral hip hardware, open fracture, active infection, severe medical comorbidity precluding anaesthesia, and inability to complete outcome assessments. No patient in either group was morbidly obese or excluded from traction-table positioning because of weight, and the two cohorts were clinically comparable in general body build.

Sample-size rationale

Kuru reported a 6.7-minute difference in operative time (standard deviation (SD) 7.5 minutes) between positions. Using this effect (d = 0.89), α = 0.05, and 90% power, 26 participants per group were required. Allowing 15% attrition, recruitment was set at 30 per group (total = 60). The study was powered for the primary endpoint (operative time); secondary outcomes were analysed with confidence intervals to reflect precision rather than powered hypotheses.​​​​​​​

Surgical procedure and perioperative care

All operations were performed by the same consultant-led team using a PFNA-II nail (200 mm, 125° caput-collum-diaphyseal (CCD)) through a mini-open approach centred on the tip of the greater trochanter. Reduction was achieved closed in every case; no open reductions or supplementary wires were used. In Group A, traction was applied through the boot; the contralateral limb was flexed and abducted to clear the image intensifier. In Group B (lateral decubitus), the entry point was obtained fluoroscopically through a small incision over the tip of the greater trochanter. The awl/guide wire was placed just medial to the trochanteric apex on the true AP view and confirmed on the lateral view before nail insertion. Counter-traction was provided by a padded longitudinal bolster and anterior iliac support. Longitudinal traction and internal rotation were delivered manually with an elastic ankle sling and maintained until locking of the jig. Rotational alignment was verified fluoroscopically; if malrotation exceeded 10°, a 3.2 mm Schanz pin joystick corrected it. Anaesthetic, antibiotic, and rehabilitation protocols were identical. Partial weight-bearing was encouraged from day 1 if pain allowed; progression to full weight-bearing depended on radiographic signs of healing.

Outcome measures

The primary outcome was operative time, defined as minutes from skin incision to skin closure, recorded with a theatre stopwatch. The secondary outcomes compared were as follows: Intraoperative metrics: (i) Set-up time: minutes from completion of final positioning to skin incision (stopwatch). (ii) Ease of fluoroscopy and (iii) Ease of traction: each graded independently by the primary surgeon and an assisting registrar immediately after skin closure on a 4-point Likert scale (1 = very difficult, 2 = difficult, 3 = easy, and 4 = very easy). For analysis, scores 3-4 were treated as “easy” and 1-2 as “difficult”; interobserver agreement was assessed and reported. (iv) Intraoperative blood loss: calculated as (suction canister volume minus total irrigation) + (swab weight gain in grams, taken as mL on a 1 g≈1 mL conversion). Post-operative radiographic quality: All measurements were performed on calibrated digital images by two blinded observers, with disagreements resolved by consensus. (i) Tip-apex distance (TAD) was measured by the Baumgaertner method on AP and lateral views and summed (mm); an accepted target range of 22-36 mm defined satisfactory placement. (ii) The Cleveland-Bosworth quadrant was recorded on AP and lateral views; central/central (C/C) or inferior/central (I/C) positions were classed as optimal. (iii) The CCD angle was measured immediately post-operatively and at four weeks; 130°-139° was considered acceptable (neutral to slight valgus). Malreduction at index surgery was defined a priori as CCD < 130° or >139° on AP, or >10° sagittal malalignment on the lateral. (iv) Fracture gap at four weeks was measured (mm) between the principal fragments at the fracture line; <5 mm was taken as acceptable. Radiographic union: signs of union were assessed at eight, 12, 16, and 24 weeks. Radiographic union was defined as a modified Radiographic Union Score for Hip (mRUSH) ≥ 10 across AP and lateral views. Functional outcome and mobilisation: Harris Hip Score (HHS) was recorded preinjury (patient recall) and at four, 12, and 24 weeks (0-100 scale; higher scores better). Post-operative day of mobilisation was documented as the first calendar day the patient stood or ambulated with aids under physiotherapy supervision. Perioperative complications (e.g., neurovascular deficit, wound complications, infection, implant failure, and reoperation) and distal neurovascular status were prospectively recorded at each visit.

Statistical analysis

Continuous variables are reported as mean ± SD or median (interquartile range) according to distribution (Shapiro-Wilk). Between-group comparisons used the independent-samples t-test, Mann-Whitney U-test, or χ²/Fisher exact test as appropriate. Interobserver reliability for Likert scales was quantified with the intraclass correlation coefficient (ICC). Two-tailed p < 0.05 signified statistical significance. All the statistical analyses were performed using SPSS v 21 (IBM Corp., Armonk, NY, USA).

## Results

Demographics and baseline characteristics

Sixty patients were included, 30 in the supine (traction table) group and 30 in the lateral decubitus group. Age, sex distribution, laterality, mechanism of injury, and fracture classifications (Boyd-Griffin and AO/OTA) were comparable between groups, and all the patients were treated with closed reduction and PFNA-II nailing (Table 1).

**Table 1:** Demographic and baseline characteristics. SD: standard deviation; I/T: intertrochanteric; AO/OTA: AO Foundation/Orthopaedic Trauma Association

Basic details	Mean ± SD/number of patients (N, %)
Operative position	
Supine	30 (50.0%)
Lateral	30 (50.0%)
Age (years)	57.23 ± 17.45
Age	
<50 years	18 (30%)
≥50 years	42 (70%)
Gender	
Male	34 (56.7%)
Female	26 (43.3%)
Mode of injury	
Road traffic accident	25 (41.7%)
Self-fall	29 (48.3%)
Hit by car/cow	6 (10.0%)
Diagnosis	
Fracture I/T femur right	33 (55.0%)
Fracture I/T femur left	27 (45.0%)
Boyd and Griffith classification	
Type 2	30 (50.0%)
Type 3	30 (50.0%)
AO/OTA classification (31A)	
31A2	30 (50.0%)
31A3	30 (50.0%)

Intraoperative parameters

**Figure 2:**
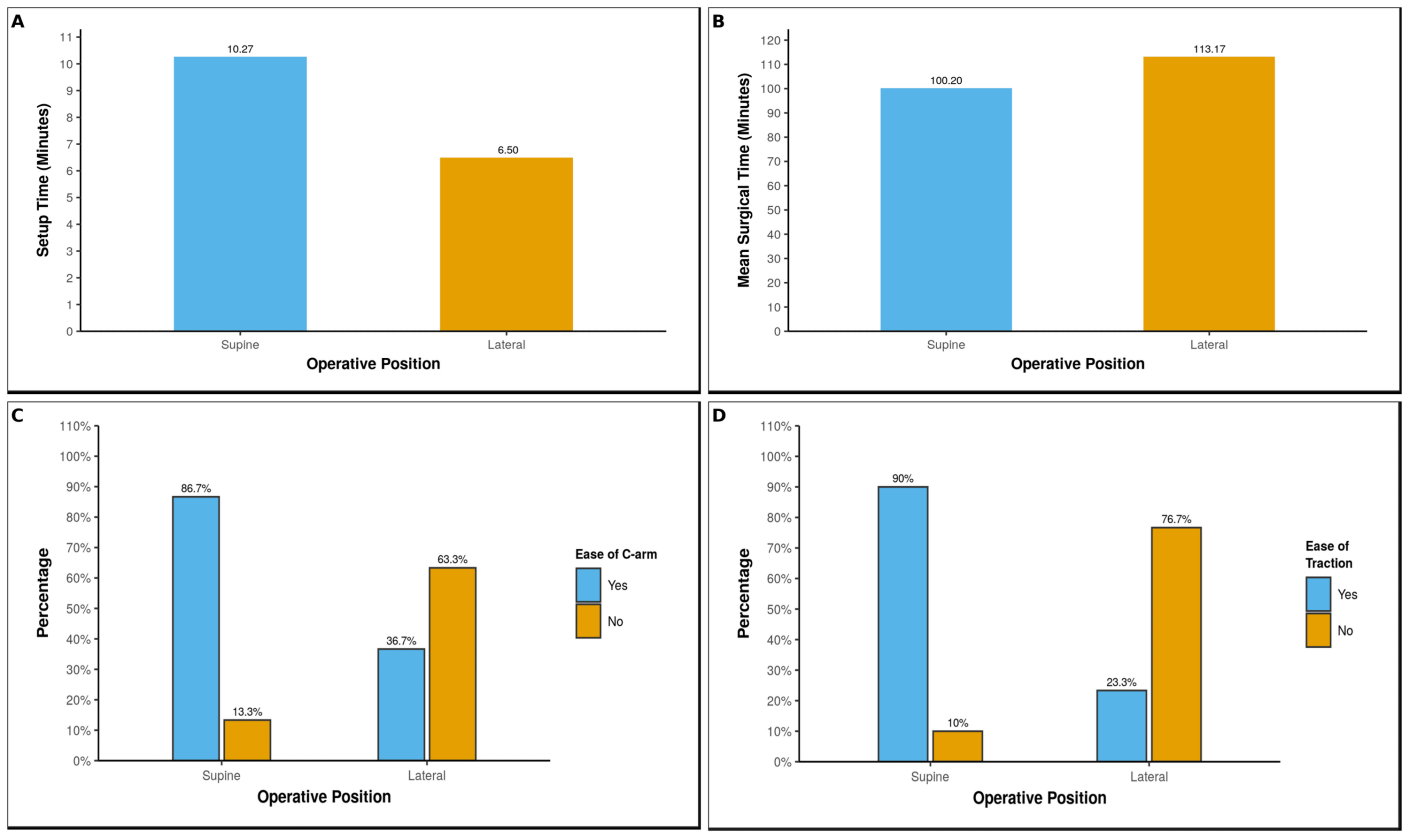
Bar graphs comparing operative efficiency and technical ease in supine vs. lateral position. (A) Set-up time was longer in the supine traction-table cohort (10.27 min) than in the lateral-decubitus cohort (6.5 min); (B) mean surgical time was shorter in the supine cohort (100.2 min) than in the lateral cohort (113.2 min); (C) ease of fluoroscopy was rated “easy” (blue) or “difficult” (orange) on the predefined 4-point Likert scale; supine positioning afforded easier image acquisition in 87% of cases; (D) ease of traction was rated “easy” or “difficult”; traction-table mechanics facilitated easy traction in 90% of supine cases, whereas manual traction was frequently challenging in the lateral position.

As shown in Figures 2A-2D, set-up time was longer for the supine cohort, reflecting traction-table preparation, whereas total surgical time was shorter than in the lateral cohort. Fluoroscopic work was consistently easier in the supine position, with more cases rated “easy” on the predefined Likert scale; traction was also more often graded “easy” when using the traction table, while manual traction in lateral decubitus required more frequent adjustments. Intraoperative blood loss was similar between groups. Observer agreement for the ease ratings was good, in keeping with the predefined methodology (Table 2).

**Table 2:** Comparison of intraoperative and postoperative parameters. Significant at p < 0.05 Statistical tests used: (1) Wilcoxon-Mann-Whitney U test, (2) Chi-squared test, (3) Fisher's exact test, (4) T-test SD: standard deviation; C/C: central/central; I/C: inferior/central; AP: anteroposterior

Parameters	Supine (n = 30)	Lateral (n = 30)	p-value
Set-up time (minutes) (mean ± SD)	10.27 ± 1.57	6.50 ± 1.22	<0.001 (1)
Mean surgical time (minutes) (mean ± SD)	100.20 ± 17.93	113.17 ± 10.64	<0.001 (1)
Ease of C-arm (yes) (n, %)	26 (86.7%)	11 (36.7%)	<0.001 (2)
Ease of traction (yes) (n, %)	27 (90.0%)	7 (23.3%)	<0.001 (2)
Intraoperative blood loss (mL) (mean ± SD)	233.50 ± 170.49	215.83 ± 152.41	0.965 (1)
Distal neurovascular deficit (n, %)			1.000 (3)
No	29 (96.7%)	30 (100.0%)	
Sciatic neurapraxia	1 (3.3%)	0 (0.0%)	
Postoperative day of mobilisation (mean ± SD)	3.43 ± 2.27	6.93 ± 4.01	<0.001 (1)
Tip-apex distance (cm) (mean ± SD)	29.17 ± 5.59	35.90 ± 7.13	<0.001 (1)
Cleveland-Bosworth quadrant (AP/lateral) (n, %)			0.11 (2)
C/C	16 (53.3%)	12 (40.0%)	
I/C	10 (33.3%)	7 (23.3%)	
Other	4 (13.3%)	11 (36.7%)	
Fracture gap (cm) (4 weeks) (mean ± SD)	2.73 ± 2.15	5.60 ± 2.18	<0.001 (1)
Collodiaphyseal angle (°) (baseline) (mean ± SD)	134.97 ± 4.13	131.70 ± 4.97	0.008 (4)
Collodiaphyseal angle (°) (4 weeks) (mean ± SD)	133.73 ± 3.64	130.27 ± 4.78	0.002 (1)

Post-operative radiographic parameters

**Figure 3:**
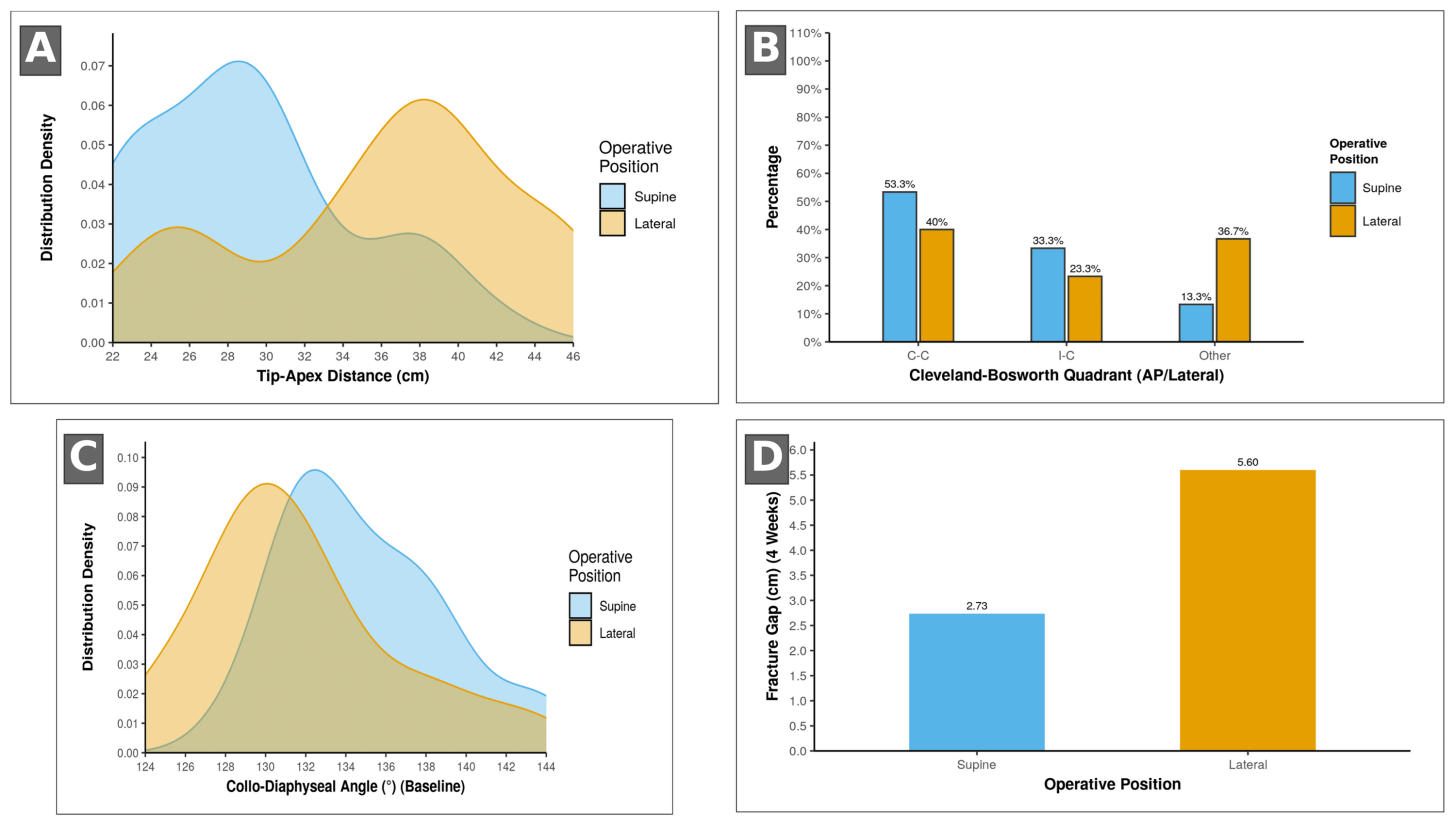
Radiographic parameters by operative position. (A) Tip-apex distance (TAD): kernel-density plot showing a lower, tighter TAD distribution in the supine group than the lateral decubitus cohort. (B) Cleveland-Bosworth quadrant: bar chart illustrating a higher proportion of helical blades in the optimal C/C (central/central) and I/C (inferior/central) zones in supine cases. (C) Collodiaphyseal angle (baseline): density curves demonstrating a more valgus-aligned reduction in supine patients compared with a modest varus tendency in lateral positioning. (D) Fracture gap at 4 weeks: column plot depicting a smaller early fracture gap in the supine group.

As shown in Figures 3A-3C, the supine group achieved more favourable implant placement. Mean TAD lay more consistently within the target range, and a higher proportion of helical blades were positioned in the Cleveland-Bosworth C/C or I/C zones. Immediate post-operative collodiaphyseal angles tended to be neutral to slight valgus in the supine cohort, whereas the lateral cohort showed a mild varus tendency. Using the a priori definition (AP CCD < 130° or >139°, or >10° sagittal malalignment), no patient met criteria for malreduction at index surgery (Table 2).

Early radiographic progression and union timeline

**Figure 4:**
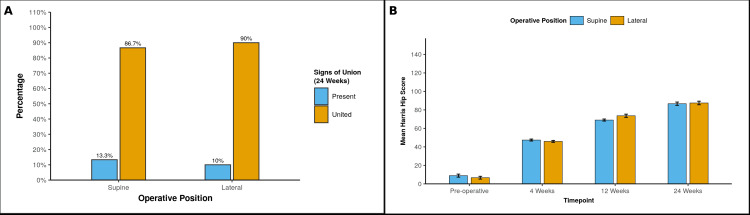
Signs of union and functional recovery in both operative positions. (A) Radiographic union at 24 weeks, which shows the percentage of fractures showing bridging callus (“Present”) versus complete union (“United”) in the supine (traction-table) and lateral-decubitus groups; (B) Mean Harris Hip score at preoperative baseline and at 4, 12, and 24 weeks post-fixation for both operative positions.

Despite the more favourable early alignment in the supine cohort, the lateral group showed earlier cortical bridging at the eight- and 12-week reviews, while the four-week fracture gap was smaller in the supine cohort (Figure 3D). By 16 and 24 weeks, radiographic union rates were comparable between groups on serial assessment using the mRUSH (Figure 4A and Table 2).

Functional outcome and mobilisation

Patients in the supine cohort commenced mobilisation sooner after surgery (first stand/ambulation day earlier), in line with the more neutral initial reductions. HHSs were similar between groups at four weeks, showed a transient advantage for the lateral cohort at 12 weeks, and converged again by 24 weeks with comparable final scores (Figure 4B and Table 3).

**Table 3:** Comparison of Harris Hip Scores between the two operative positions. Score mentioned as mean ± SD SD: standard deviation

Parameters	Supine (n = 30)	Lateral (n = 30)	p-value
Harris Hip Score (preoperative)	8.91 ± 8.91	6.68 ± 8.32	0.394
Harris Hip Score (4 weeks)	47.41 ± 5.79	45.95 ± 5.69	0.129
Harris Hip Score (12 weeks)	69.03 ± 6.12	73.67 ± 9.46	0.029
Harris Hip Score (24 weeks)	86.71 ± 9.69	87.50 ± 10.61	0.818

Complications

Perioperative complications were infrequent. One patient in the supine group developed a transient sciatic neurapraxia that resolved without intervention. Minor serous wound discharge occurred in a small number of cases and settled with local care and antibiotics; there were no deep infections. Delayed union was recorded in a few patients in each cohort, with no implant failures or re-operations during the 24-week follow-up (Table 2).

## Discussion

This prospective comparison of supine traction-table versus lateral decubitus positioning for proximal femoral nailing in unstable intertrochanteric fractures found that operative position influenced several intraoperative steps and early radiographic parameters but had little effect on final union or 24-week function. Supine positioning required a longer set-up yet enabled shorter operative time, easier fluoroscopy and traction, smaller early fracture gaps, more neutral/valgus collodiaphyseal alignment, and more consistent implant placement (lower mean TAD and C/C or I/C position). Lateral positioning showed a tendency to earlier cortical continuity at 8-12 weeks, but union rates and HHSs converged by 16-24 weeks. Complications were infrequent in both cohorts.

During supine positioning, the use of a traction table prolonged preparation by roughly four minutes because the foot boots and perineal post had to be mounted, and the contralateral limb had to be flexed and abducted. Once draping was complete, however, mechanical traction held a stable reduction and freed the assistant to concentrate on guide-wire alignment; operative time was therefore 13 minutes shorter than in the lateral cohort. This reduces radiation exposure and anaesthetic duration-advantages that may appear modest but accumulate in busy trauma lists and are consistent with findings from other femoral-nailing studies that compared operative positions [9,10]. Intraoperative blood loss did not differ between groups, indicating that incision length and reaming technique, rather than position, dictate blood loss volume.

Controlled traction and an unobstructed AP C-arm arc allowed more accurate implant placement in the supine cohort. The mean TAD of 29 mm in the supine group remained below the critical 30 mm threshold, while 87% of helical blades lay in the C/C or I/C Cleveland-Bosworth zones. A traction-table corridor also permitted a slightly more medial “bald-spot” entry, which, together with a neutral-to-valgus CCD angle, prevents against varus collapse and cut-out described in the literature [11,12]. In lateral decubitus, the femur is elevated from the table, and the image intensifier must swing under the hip or be rotated like an umbrella; this complicates true AP fluoroscopy and explains the broader TAD and less favourable entry alignment observed with lateral position [13,14].

The relationship between early fracture gap and subsequent cortical bridging deserves careful interpretation. In the supine cohort, the traction table allowed sustained reduction, controlled impaction, and more accurate implant placement, which likely contributed to the smaller fracture gap seen at four weeks. In contrast, the lateral cohort demonstrated earlier cortical bridging at eight and 12 weeks despite a slightly larger early gap. A plausible explanation is that fixation after manual traction release may have allowed a small degree of controlled interfragmentary motion, thereby promoting periosteal callus formation. This would explain why early bridging was more apparent in the lateral group, even though the immediate mechanical reduction was less favourable. The convergence of union rates by 16 and 24 weeks suggests that these differences represent variation in the early mode of healing rather than a true difference in eventual union [15]. By 16 weeks, union rates were comparable, similar to the temporal pattern reported by Xue et al. and confirming that either set-up supports reliable healing [16,17].

Earlier mobilisation in the supine group likely reflects the more controlled reduction, smaller early fracture gap, and more favourable implant alignment achieved with traction-table assistance. Together, these factors may provide greater confidence in early stability and permit a more liberal mobilisation strategy, especially in elderly patients at risk from prolonged immobilisation. Nevertheless, the lateral cohort had caught up by 24 weeks, yielding equivalent HHSs; a similar finding was described by Ozkan et al. [18]. Body habitus was unlikely to have confounded this finding, as patients in both cohorts were of comparable weight range and no obese or morbidly obese patients were included.

There were only a few complications. One transient sciatic neurapraxia occurred in a traction-table case-a recognised risk when the post compresses the gluteal region [19,20]. No malreductions or implant failures were recorded, stating that both positions are safe when meticulous technique is applied.

These observations suggest a pragmatic algorithm. Where fracture tables and trained staff are available, supine positioning offers better reduction, optimal blade placement, and quicker rehabilitation-attributes especially valuable in elderly, comorbid patients, at risk of pulmonary or thrombo-embolic events. Lateral decubitus remains indispensable when traction equipment is unavailable, when perineal-post complications are a concern, or when spinal precautions preclude supine positioning. Ultimately, success lies less in the operative position than in achieving a balanced reduction, a C/C blade with acceptable TAD, and prompt mobilisation.

Limitations

This was a single-centre, prospective comparative cohort with non-random allocation determined by intraoperative set-up availability, introducing potential selection bias and unmeasured confounding. We mitigated this by prespecifying inclusion/exclusion criteria, using a uniform implant and perioperative protocol, and blinding radiographic assessors. Analytically, we verified that baseline characteristics were similar and performed adjusted models for key outcomes, which did not materially change the estimates. Future randomised or propensity-matched multicentre studies are warranted. Subjective ratings of fluoroscopy and traction ease may introduce observer bias, yet good interobserver reliability (ICC > 0.79) supports their consistency. The study was powered for operative-time differences, not for secondary endpoints such as Harris scores or early union; those findings, while clinically plausible, should be interpreted cautiously. Follow-up at 24 weeks might miss late failures, although most mechanical complications of PFNA occur earlier. Body weight was not analysed as an independent variable. Although no morbidly obese patient was included, obesity can influence the feasibility of traction-table positioning and may affect the generalisability of these findings to heavier patient populations. Despite these limitations, the present work provides prospective data and helps to clarify where each operative position provides genuine value.

## Conclusions

In this prospective comparison of operative positions for proximal femoral nailing in unstable intertrochanteric fractures, both supine on a traction table and lateral decubitus achieved similar union by 16-24 weeks and comparable 24-week function. Supine positioning delivered clearer fluoroscopic access, steadier traction, more consistent implant placement (lower TAD, C/C or I/C Cleveland-Bosworth zones, and neutral-valgus collodiaphyseal angles), and earlier mobilisation. Lateral decubitus remained safe and effective, with slightly earlier callus on radiographs in some patients, but tended to mobilise later; blood loss and complication rates were low and similar overall. When a traction table and trained staff are available, supine positioning is a practical default for unstable patterns because it simplifies reduction and imaging and supports earlier mobilisation-advantages that matter in frail older adults. Lateral positioning is a sound alternative where traction tables are unavailable or undesirable (e.g., to avoid perineal-post complications, in polytrauma/spinal precautions). Position influences intraoperative ease and early recovery more than it does final healing or function. Surgeons should select the position that best matches theatre resources and patient needs, confident that either approach yields reliable healing when sound reduction and nail placement are achieved.
